# stLFRsv: A Germline Structural Variant Analysis Pipeline Using Co-barcoded Reads

**DOI:** 10.3389/fgene.2021.636239

**Published:** 2021-03-18

**Authors:** Junfu Guo, Chang Shi, Xi Chen, Ou Wang, Ping Liu, Huanming Yang, Xun Xu, Wenwei Zhang, Hongmei Zhu

**Affiliations:** ^1^BGI-Tianjin, BGI-Shenzhen, Tianjin, China; ^2^BGI-Shenzhen, Shenzhen, China; ^3^MGI, BGI-Shenzhen, Shenzhen, China; ^4^Guangdong Provincial Academician Workstation of BGI Synthetic Genomics, BGI-Shenzhen, Shenzhen, China; ^5^Guangdong Provincial Key Laboratory of Genome Read and Write, BGI-Shenzhen, Shenzhen, China

**Keywords:** **:** human genome, co-barcoded reads, structural variation, complex variants, breakpoints

## Abstract

Co-barcoded reads originating from long DNA fragments (mean length >30 kbp) maintain both single base level accuracy and long-range genomic information. We propose a pipeline, stLFRsv, to detect structural variation using co-barcoded reads. stLFRsv identifies abnormal large gaps between co-barcoded reads to detect potential breakpoints and reconstruct complex structural variants (SVs). Haplotype phasing by co-barcoded reads increases the signal to noise ratio, and barcode sharing profiles are used to filter out false positives. We integrate the short read SV caller smoove for smaller variants with stLFRsv. The integrated pipeline was evaluated on the well-characterized genome HG002/NA24385, and 74.5% precision and a 22.4% recall rate were obtained for deletions. stLFRsv revealed some large variants not included in the benchmark set that were verified by long reads or assembly. For the HG001/NA12878 genome, stLFRsv also achieved the best performance for both resource usage and the detection of large variants. Our work indicates that co-barcoded read technology has the potential to improve genome completeness.

## Introduction

Structural variants (SVs) represent genome variants larger than 50 bp consisting of deletions, insertions, inversions, duplications, and translocations (Feuk et al., 2006; Alkan et al., 2011). SVs contribute more genomic sequence differences than single-nucleotide polymorphisms (SNPs) or small indels between genomes (Pang et al., 2010). Some of these SVs are pathogenic variants associated with specific diseases (Singleton et al., 2003; Jongmans et al., 2006; Rovelet-Lecrux et al., 2006). Despite the importance of SVs, profiling them has been challenging.

For the last 20 years, several technologies have allowed SV annotation to improve and have helped to generate a well-characterized human genome reference sequence to facilitate the development of SV identification tools (Zook et al., 2019[Preprint]). Among these technologies, sequencing is a primary category that includes long read, short read, and co-barcoded read sequencing. Each sequencing technique has unique advantages and disadvantages that contribute to the discovery of SV profiles among populations.

Long reads or single-molecule sequencing reads usually have mean length greater than 10 kbp. These longer reads identify breakpoints more easily and may span nearby repetitive regions of several kilobases (Jain et al., 2018). However, long reads are prone to insertion and deletion errors, and the base level accuracy is comparatively low, which leads to low accuracy for small variant (less than 200 bp) detection (Wang et al., 2019). The single-molecule circular consensus sequencing protocol, which improves base level accuracy, produces high-quality reads that average >10 kbp (Wenger et al., 2019). However, this protocol is not applicable to large-scale projects because of throughput and cost limitations.

Short reads are accurate at the base level and cost-effective. Their uniform depth and insert size can be successfully used to identify deletions and copy number variation (Layer et al., 2014; Talevich et al., 2016). Deletions are easier to detect than insertions. However, more complex variants are rarely detected with short reads because their breakpoints are usually in close proximity to regions lacking unique short read alignment.

To compensate for the lack of long-range information, co-barcoded read sequencing was developed. Co-barcoded reads are the product of novel protocols for library construction and next-generation sequencing (NGS) sequencing technology. There are two mature technologies in this category, Linked-Reads by 10× Genomics (Zheng et al., 2016; Zhang et al., 2017) and single-tube long fragment read (stLFR) by BGI (Wang et al., 2019). In both cases, all the short reads that originate from the same long DNA molecule will share a common barcode. Thus, they retain long-range genome information while maintaining base level accuracy. Only nanograms of input DNA is needed, making co-barcoding feasible for many applications. The inferred average DNA fragment length for co-barcoded reads is approximately 30 kbp, which makes it possible to sequence across even larger repetitive regions near SV breakpoints. stLFR uses a combinatorial process to generate up to 3.6 billion unique barcodes, enabling practically nonredundant co-barcoding with 50 million barcodes per sample. Compared with Linked-Reads, stLFR can achieve a much lower barcode conflict rate (how many long DNA molecules share one barcode), which is beneficial for downstream analyses.

Analysis pipelines that detect SVs with co-barcoded reads fall into three categories based on how they use barcode information. The first category identifies novel adjacency by detecting abnormal numbers of common barcodes shared between two genomic loci or bins (Spies et al., 2017; Xia et al., 2018; Marks et al., 2019). The second tests the distribution of sequenced short segments on large DNA molecules (Elyanow et al., 2018; Marks et al., 2019). The third uses barcode information to extract data for local assembly (Meleshko et al., 2019[Preprint]; Zhou et al., 2019[Preprint]).

Here, we present stLFRsv, a co-barcoded read-based SV analysis pipeline that falls into the first category and integrates the short read SV detector smoove (Brent, 2018).

## Methods

**FIGURE 1:**
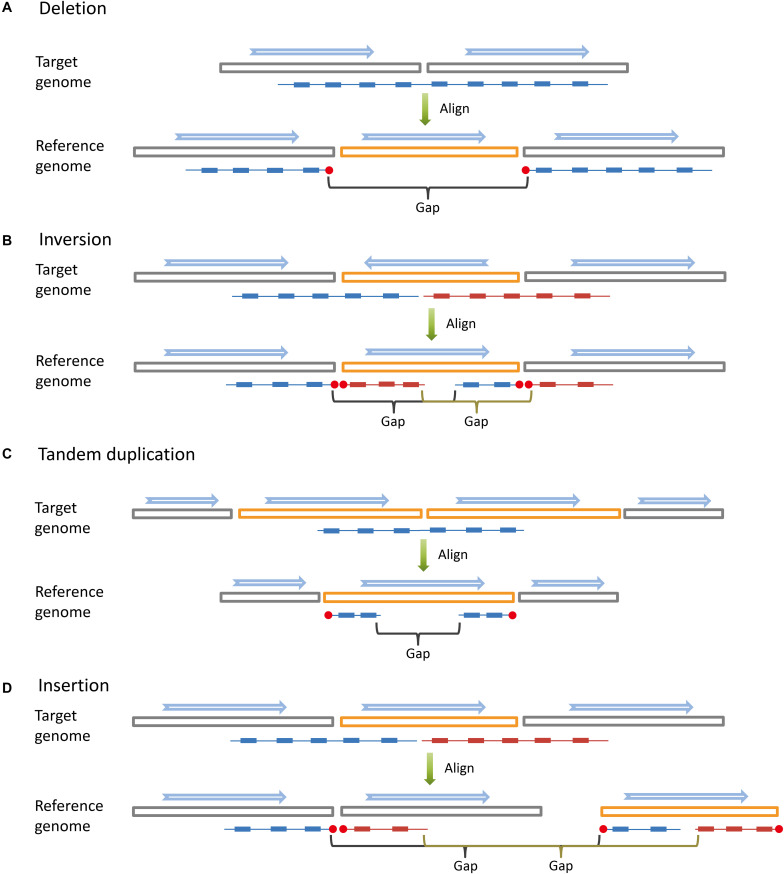
Long DNA fragments (colored lines) are constructed by read pairs (small solid blocks) that share the same barcode. When aligned to the reference genome, long DNA fragments covering large structural variations are broken into sub-fragments by large gaps. The blue arrows indicate the directions of genome sequences (big hollow blocks). **(A)** Deletion. **(B)** Inversion. **(C)** Tandem duplication. **(D)** Insertion.

**FIGURE 2:**
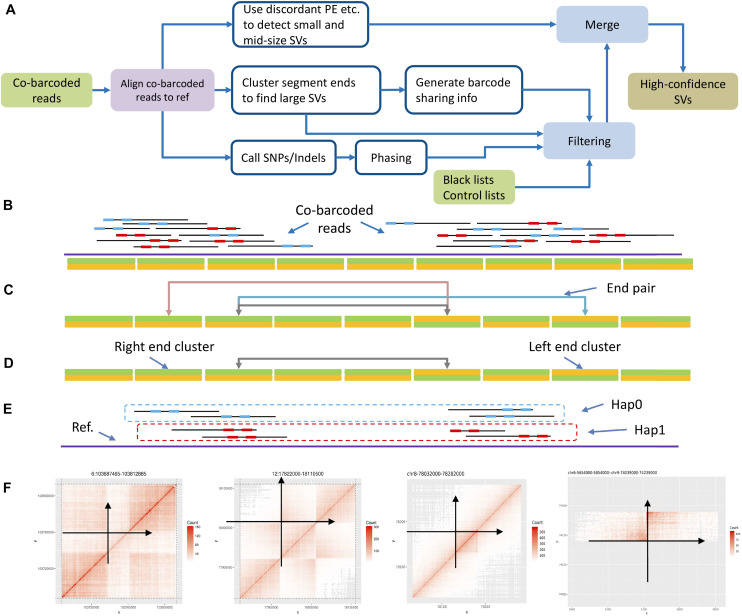
Workflow and algorithm. **(A)** Structural variation detection workflow. **(B)** Cluster segment ends by bins: left end cluster and right end cluster. **(C)** Pair up ends by shared barcodes. **(D)** Pair down candidates by removing those nearby. **(E)** Split into haplotypes by phasing barcodes on phasing blocks. **(F)** Use barcode sharing heatmap pattern as a filter, and anchor the variation on the genome. Each point in the heatmap represents the shared barcode number at the corresponding *X*-axis and *Y*-axis positions by color depth.

Large SVs leave apparent large gaps in long fragments based on co-barcoded read alignment (Figure 1). The distribution of read pairs on long fragments is approximately random, and the gap sizes between read pairs vary in a wide range. Large gaps appear in long fragments by chance. However, large SVs are likely to lead to large gap aggregation. Thus, stLFRsv detects large gaps in fragments to identify large variants. In contrast, smoove is a pipeline that uses LUMPY as its core to detect paired-end discordance and other short read signals that indicate variants (Layer et al., 2014). We use smoove to find small and mid-sized variants. There may be overlap between the two variant sets, and thus, we merge them before generating the results (Figure 2A). The detection process for stLFRsv is described in the following steps.

### Cluster Segment Ends

We calculate an empirical gap size distribution and select a size *G* as cut-off such that the probability of gap sizes smaller than *G* is *P* (Supplementary Figure 2). Usually, *P* is set as 98%, which is reasonable based on statistics. When we break a long fragment at a gap larger than *G*, we get two sub-fragments. We define the starting and terminal positions of a sub-fragment as the left and right ends. Each end has its position on the reference. We then divide the reference sequence into consecutive bins. Each bin has a size of *B bp* based on the data profile, which holds left and right ends and serves as left and right end clusters. Additionally, *B* is selected in the same way as *G* with *P* set to 65%, which aims to achieve a fine cluster performance and maintain reasonable precision for end positions. All end clusters with at least one end are retained for the next step (Figure 2B).

### Pair Up Ends

Every two end clusters are checked for common barcodes to determine whether they could form a high-quality end pair (Figure 2C). These end pairs with common barcodes are potential novel adjacencies and are further checked as follows. First, the sub-fragment lengths for each barcode are collected to estimate the probability *f*(*d*) that one barcode is observed at both locations *locA* and *locB* with a distance of *d*. *f*(*d*) is defined as follows:

$$f\,\left(d\right)=\sum_{l>d}P\,\left(l\right)*\frac{l-d}{l+B}$$

in which *l* is the sub-fragment length, *P*(*l*) is the probability of length *l*, and *B* is the size for clustering mentioned above. Second, the high-quality end pairs with a distance of *d* are decided by the following three rules. (1) The number of shared barcodes of two end clusters is higher than the theoretical value calculated by *f*(*d*). (2) The barcode counts of each end cluster are *N* standard deviations higher than average depth (using *N* = 3 by default in the pipeline). (3) The barcode counts of each end cluster are significantly higher than neighboring clusters with *P*-values less than *p_th* by Wilcoxon signed-rank test (using default *p_th* = 0.1 in the pipeline).

There are four types of end pairs according to the types of the two end clusters. If the potential novel adjacency does not involve an orientation change, the end pair is a right–left or left–right. Otherwise, it is either a left–left or right–right type (Figure 1). If an end cluster is in pair with multiple clusters and one of the pairs is very likely to be the two ends of a sub-fragment, we unpair them.

### Pair Down Candidates

Because the DNA molecules are partially sequenced, sub-fragment ends do not gather densely around a novel adjacency. They may spread in several bins and give rise to multiple end pairs. According to the gap size distribution mentioned above, a size of *Nmerge* is chosen with *P* set to 93%. To reduce redundancy, for each end pair, we recursively compare its common barcode number with that of pairs in the same type within a range of *Nmerge*, retain a representative end pair with the highest common barcode number, and refine the positions (Figure 2D).

### Split by Haplotypes

Approximately 60% of reads can be haplotype solved, which means that those reads along with their barcodes are placed onto one of the haplotypes of each phasing block (Figure 2E). Thus, the merged end pairs are checked and screened by the phasing info of their common barcodes. First, each end pair is assigned to a haplotype according to the haplotype of the common barcodes. The end pair without sufficient phased common barcodes will be assigned to one haplotype randomly. Then, the end pairs assigned to the same haplotype and sharing the same end cluster are gathered and sorted by the number of common barcodes in descending order. Finally, only the pair with the most common barcodes will be kept, because for one end cluster, a true novel adjacency only forms one end pair on the same haplotype.

### Filter

Noisy signals often result in false novel adjacencies. The following noise filters can mitigate this problem.

#### Common Barcode Heatmap

The first filter uses the common barcode heatmap around each novel adjacency region (Figure 2F). A novel adjacency increases the number of common barcodes. This increase shows specific patterns in the regions in close proximity to the novel adjacency on the heatmap. Because this is not a graphic detector, we digitize the heatmap to reveal patterns. Horizontal and vertical directions intersect at the breakpoints on the heatmap, which forms four regions. For a deletion, insertion, or duplication, there is only one region showing typical adjacency barcode sharing. For an inversion, there are two regions with symmetric sharing. We collect bin-to-bin barcode sharing numbers in each region and use the Wilcoxon signed-rank test to verify the expected patterns between each two of the four regions.

#### Common Barcode Phase

This filter uses the phase info of the common barcodes. For each novel adjacency, if the proportion of phased common barcodes is greater than 75%, the numbers of barcodes phased to each of the two haplotypes are checked using Fisher’s exact test against ideal (1|0), (0|1), and (1|1) zygosity cases. For a true novel adjacency, only one case should be significantly matched with a distinct *P*-value.

#### Anchor the Breakpoints

If a novel adjacency is formed by a pair of ends that are distant from each other on the reference, we would like to know whether this rearrangement results in a short interruption or a long-range SV.

Due to the limited DNA fragment lengths, the numbers of shared barcodes decrease gradually in bins further from the novel adjacency. When end pairs are placed on the target genome, they all present as a left end and a right end. If we check the common barcode numbers between the bin holding the right end and the bin holding the left end and each of the bins following the left, the common barcode numbers should show a gradual decline. We calculate the fading rates and the counts by which the observed numbers exceed the expected numbers according to the distribution *f*(*d*) described above. The process is similar to that of the left end. For each end pair, we have two lists of deviations and fading rates. The end pairs are then tested by a Wilcoxon signed-rank test to detect the asymmetry of fading in both directions and a sudden loss of barcode sharing in one direction. If there is evidence of asymmetry or short-range extension, we infer that a short sequence from a distance was inserted into one direction and assign a low confidence score. Otherwise, a high confidence score is assigned. This estimation is more accurate for haplotype-solved novel adjacencies.

#### Map Quality

The read mapping qualities are checked within the range of *Nmerge* around the two ends of each pair, and the pairs are screened out if the low-quality ratio is above a set cut-off. A low confidence score will be given if the percentage of reads with low mapping quality is greater than 50.

#### Read Pairs

For regular paired-end sequencing data, the insert size of a read pair is important evidence for SV detection. The read pairs with an abnormal insert size are also checked for a novel adjacency. There is a corresponding relationship between the adjacency end orientation and the paired-end map orientation: right–left vs. forward–reverse, left–right vs. reverse–forward, left–left vs. reverse–reverse, and right–right vs. forward–forward. Four types of abnormal read pairs are counted to evaluate whether they match or conflict with the adjacency type. Additionally, if there is a match, the resolution of the adjacency will be refined from an *Nmerge* size to a normal paired-end insert size.

#### Black and Control Lists

Candidate pairs are filtered out in the problematic regions of the reference. These regions are defined as *black regions*, which are formed based on the reference profile and usually involve repeat sequences, mis-assembled areas, and gaps. Moreover, another set of regions defined as *control regions* is also used to filter the candidates. The *control regions* contain segmental duplications, high population frequency, and other systematic SV regions caused by the aligner, sequencer, library method, etc.

Finally, a comprehensive confidence score is generated based on the confidence scores from the filters. Then the adjacencies with high comprehensive confidence scores will be passed to downstream steps.

### Merge

We extract variants below a cut-off size from smoove results and those above this cut-off from stLFRsv results and combine them by merging those with significant overlap (at least 70% overlap with respect to the longer SVs) to form the final output.

## Results

### stLFR Co-barcoded Read Data of HG002

#### Data Preparation

The HG002 cell line sample was processed according to the stLFR protocol (Wang et al., 2019) and sequenced to 100× coverage. The average number of read pairs per barcode was 51. The inferred weighted fragment length was 83 kbp. The inferred mean number of fragments per barcode was 1.15. The distributions of read pair numbers, weighted fragment lengths, and fragment number per barcode are illustrated in Supplementary Figure 1. We down-sampled the data to 50× and 30× and called variants separately to provide guidance for applications. stLFRsv was assessed on the HG002 genome in manual parameter mode against the following four SV callers: Long Ranger, NAIBR, smoove, and GROC-SVs (Spies et al., 2017; Elyanow et al., 2018; Marks et al., 2019). The results from co-barcoded reads were also compared with SVs from 100× Nanopore long reads. The commands used to run the following pipelines are shown in Supplementary Table 1.

#### Structural Variation

The workflow of structural variation detection is illustrated in Figure 2A. Co-barcoded reads were aligned to hs37d5 by BWA-MEM2 (Li, 2013[Preprint]; Vasimuddin et al., 2019). Phasing was performed by HapCUT2 after SNPs were called using GATK (McKenna et al., 2010; Edge et al., 2017). The GIAB v0.6.2 structural variation set includes 7,172 insertions and 5,336 deletions. We used Truvari to align pipeline calls to the GIAB call set^1^. For Long Ranger, the alignment was performed by Lariat. For other software, the alignment results by BWA-MEM2 were used.

**FIGURE 3:**
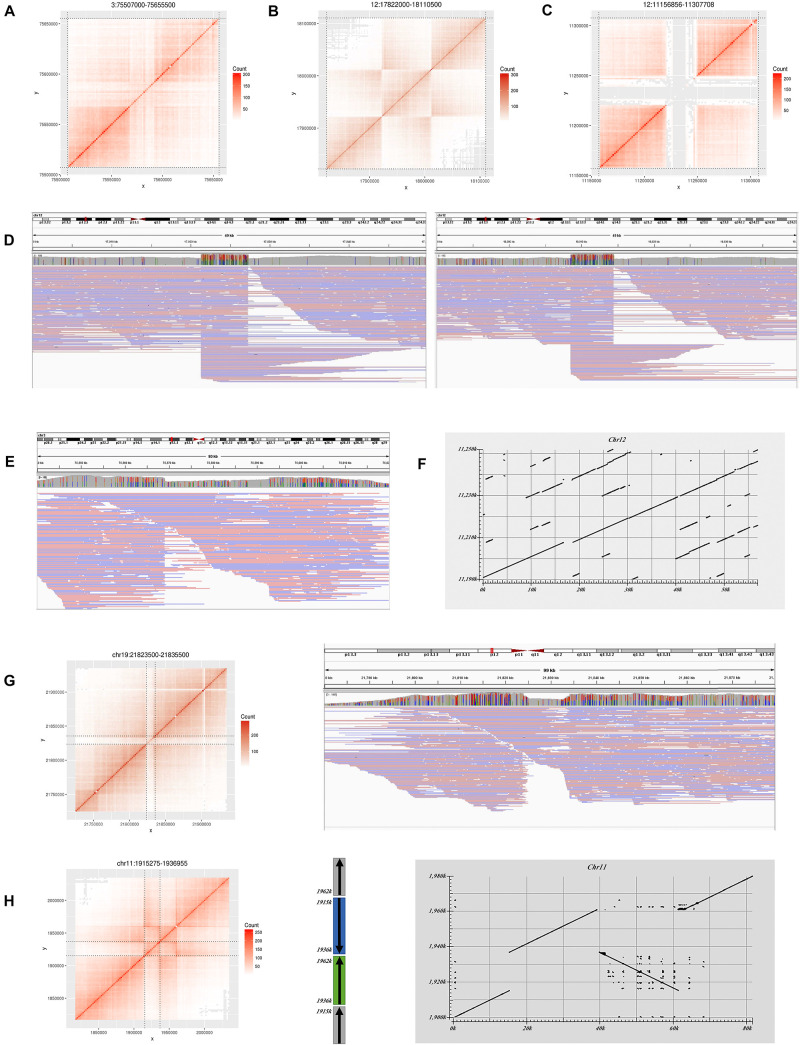
Large variations do not match the GIAB benchmark in HG002. **(A)** Heatmap for a deletion on Chr3. **(B)** Heatmap for an inversion on Chr12. **(C)** Heatmap for a deletion on Chr12. **(D)** Long read alignment supports the inversion in **(B)**. **(E)** Long read alignment supports the deletion in **(A)**. **(F)** Assembly alignment to reference by Blast for the deletion in **(C)**. **(G)** Heatmap for a deletion on Chr19 and long read alignment. **(H)** Heatmap and structure for an inversion on Chr11 and assembly alignment.

Seventy-nine large deletions were identified by stLFRsv. Thirty-seven of these were validated by the GIAB call set with the quality flag “PASS.” Among the 42 unmatched deletions, 12 overlap with the GIAB deletion records but were failed by Truvari because of the overlap ratio. Twenty-six of the unmatched deletions overlap with the GIAB deletions with markers other than “PASS” (Supplementary Table 2). One is located at Chr12:11,216,856–Chr12:11,247,708 (Figure 3C). Several confusing signals were observed at the start of this deletion in both the co-barcoded reads and Nanopore long-read mapping results. Thus, the Nanopore assembly sequence was compared with the reference sequence. The result shows that there are two approximately 20 kbp segment duplications near the start and the end of this region. The downstream region is highly matched with the *hs37d5* decoy sequence, which explains the detection of this deletion (Figure 3F).

Four deletions do not overlap any GIAB record. Two were marked with “COMMON” by the *control list*, and the other two were marked with “PASS.” Only the “PASS” two were confirmed in the Nanopore long reads results. One is located at Chr3:75,567,000–75,595,500 and supported by Nanopore long reads (Figures 3A,E). The other is located at Chr19:21,822,000–21,835,500 and inferred as a heterozygous variant by long reads (Figure 3G).

Only three GIAB deletions larger than 10 kbp were not detected by stLFRsv, and the heatmaps for these deletions are shown in Supplementary Figure 3A. Two of these are in the N-regions of the reference on ChrX, and they were filtered out by the *Black list*. The third deletion is a heterozygous deletion and was undetected because the length of the DNA fragment between this deletion and the following homozygous deletion is too short for co-barcode SV detection.

In addition to deletions, stLFRsv identified 55 inversions, duplications, and translocations (Supplementary Table 3). Most of these are shared by multiple genomes, which indicate problematic reference regions or repeat sequences on the reference and were marked on the *Control list*. Some of them are caused by the alignment characteristics of short reads that could not be confirmed by Nanopore long reads. Others may indicate the difference between the reference and the population. For example, two inversions were also observed in HG001/NA12878 and some other samples. One has a typical inversion structure on the heatmap and was found at Chr12:17,922,000–Chr12:18,013,500 (Figures 3B,D). It was classified to be a homozygous variant and confirmed by Nanopore reads. The other has a more complex dual-inversion structure in which a sub-fragment of an inverted fragment reversed again and was confirmed by long read assembly (Figure 3H).

Furthermore, deletions (>10 kbp) that were not detected by stLFRsv but were detected by other co-barcoded read-based SV callers are listed in Supplementary Table 4. There are 40 deletions in total, 12 from Long Ranger, 5 from GROC-SVs, and 23 from NAIBR. Approximately 50% of these deletions were observed in stLFRsv but were filtered by the region filter (*Black list*). None were validated by the GIAB call set with a quality flag “PASS” except the three deletions mentioned above (Supplementary Figure 3A). Twenty-eight of these deletions are likely the result of improper short read alignments, and another eight do not overlap with GIAB call set records. One deletion at Chr8:8,032,452–Chr8:8,045,361 was chosen to evaluate the difference between the regular aligner BWA-MEM2 and the co-barcode aware aligner Lariat (aligner of Long Ranger pipeline). As shown by the heatmaps in Supplementary Figure 3B, although the improper alignments causing a deletion call in a complex region were corrected to a certain degree, Long Ranger still marked it as a reliable deletion. Despite its preferable performance in NGS “dead zone” genes (Mandelker et al., 2016; Marks et al., 2019), the co-barcode-aware aligner does not seem to provide significant improvements on large and complex genomic regions.

When merging deletions from stLFRsv and smoove, the size cut-off was set to 10 kbp by stLFRsv based on the data profiles. The deletion evaluation results are shown in Table 1. The down-sampled results are in Supplementary Table 5. Because few insertions were found by any of the four callers, we did not evaluate insertion results.

**TABLE 1:** Deletion evaluation on whole genome against GIAB HG002 benchmark.

		100× long reads	100× co-barcoded reads					
	
		Sniffles	Long Ranger	NAIBR	stLFRsv	smoove	stLFRsv + smoove	GROC-SVs
	Mapping	Minimap2	lariat	bwamem2	bwamem2	bwamem2	bwamem2	bwamem2
50–1 k	Benchmark	4,719						
	Total call	9,453	3,583	2	0	972	972	0
	TP	4,168	2,304	2	0	724	724	0
	FP	5,285	1,279	0	0	248	248	0
	FN	551	2,415	4,717	4,719	3,995	3,995	4,719
	Precision	44.09%	64.30%	100.00%	–	74.49%	74.49%	–
	Recall	88.32%	48.82%	0.04%	–	15.34%	15.34%	–
1 k–10 k	Benchmark	577						
	Total call	902	489	155	13	554	556	0
	TP	533	391	125	12	434	436	0
	FP	369	98	30	1	120	120	0
	FN	44	186	452	565	143	141	577
	Precision	59.09%	79.96%	80.65%	92.31%	78.34%	78.42%	–
	Recall	92.37%	67.76%	21.66%	2.08%	75.22%	75.56%	–
10 k–30 k	Benchmark	31						
	Total call	60	27	31	56	35	56	9
	TP	28	19	24	30	22	30	7
	FP	32	8	7	26	13	26	2
	FN	3	12	7	1	9	1	24
	Precision	46.67%	70.37%	77.42%	53.57%	62.86%	53.57%	77.78%
	Recall	90.32%	61.29%	77.42%	96.77%	70.97%	96.77%	22.58%
>30 k	Benchmark	9						
	Total call	55	14	28	23	36	23	13
	TP	9	6	8	7	7	7	4
	FP	46	8	20	16	29	16	9
	FN	0	3	1	2	2	2	5
	Precision	16.36%	42.86%	28.57%	30.43%	19.44%	30.43%	30.77%
	Recall	100.00%	66.67%	88.89%	77.78%	77.78%	77.78%	44.44%

Unlike stLFRsv, Long Ranger, and GROC-SVs combine the co-barcode information with a local assembly strategy, which enables them to detect SVs around short sequences with high-quality alignments, such as the deletion on Chr2 shown in Supplementary Figure 3A, but with lower sensitivity. In contrast, NAIBR is based on a model using paired-end discordance along with co-barcode information. This model leads to higher sensitivity, especially for SVs with small size or around N-regions, such as the deletions on ChrX shown in Supplementary Figure 3A, but it also suffers from more false-positive SVs.

#### Testing Built-in Parameter Setting on Multiple HG002 Libraries

If not specified, stLFRsv offers an auto parameter mode to estimate parameters according to the following data profiles: distribution of DNA fragment length and inter-read-pair gap length. As mentioned in section “Methods,” “Large-gap” size *G* to break fragment into sub-fragment, bin size *B* to cluster sub-fragment borders, and merging size *Nmerge* to merge bins into a single breakpoint are chosen based on inter-read-pair gap length distribution. These three parameters then determine the sensitivity of the pipeline and the accuracy of the breakpoint locations. In contrast, the sizes of inversion and duplication that stLFR is able to identify are dictated by the DNA fragment length distribution. Long DNA fragments only detect large inversions and duplications. The detectable deletion size should be larger than the “large-gap” size *G*.

For the HG002 cell line sample, we constructed four stLFR libraries to assess the influence of the data profile. Only high-quality reads (>4 read pairs per segment and >8 read pairs per barcode) were retained for statistical analysis. The data statistics and inferred parameters for these four HG002 libraries are illustrated in Table 2 and Supplementary Figure 4. It is highly recommended, according to our tests, not only for stLFRsv but also for other co-barcoded read-based SV callers that stLFR data should have a high-quality read ratio >70%, average read pairs per segment >25, and barcode conflict <1.7 for good detection performance.

**TABLE 2:** Detection capability and estimated parameters of different HG002 libraries.

**Library**		**HG002-1**	**HG002-2**	**HG002-3**	**HG002-4**
Input DNA amount		1 ng	1 ng	1.5 ng	1.5 ng
Reads count		2,525,286,352	3,029,968,430	2,172,780,252	2,994,596,020
Average sequencing depth (after duplication removed)		44.34	35.77	46.73	44.38
High-quality read ratio		89.57%	78.03%	79.15%	75.55%
Read pairs per segment		32.33	18.30	18.40	17.21
Barcode conflict (segments per barcode)		1.55	1.41	2.04	1.70
Estimated parameters	*B* (bp)	1,500	1,500	2,500	1,900
	*Nmerge* (*B*)	4	4	4	4
	*G* (bp)	13,100	13,900	22,200	13,800
Detection capability	Deletion (bp)	13,500	13,500	22,500	13,300
	Inversion/duplication (bp)	48,100	28,600	46,700	32,200

#### Comparison With Nanopore Long Reads

We obtained 100× Nanopore long reads of HG002 from Oxford Nanopore Technologies. The distribution of read length and percent identity are presented in Supplementary Figure 1. The alignment was performed with Minimap2 using default parameters (Li, 2018). SVs were detected by Sniffles with default parameters, and increasing the support read number can reduce both false positives and true positives (Sedlazeck et al., 2018). The deletion evaluation is also listed in Table 1. The insertion evaluation is shown in Supplementary Table 6. We assembled long reads by NECAT for variation validation (Chen et al., 2020).

For deletions, Nanopore long reads achieve a high sensitivity in every size level along with a number of false-positive deletions. stLFRsv attains approximately the same level of sensitivity with a lower false-positive rate for large deletions. For insertions, Nanopore long reads show the same performance as deletion detection with small insertions but fail for large insertions just like stLFR and the other three SV callers. This result is consistent with a previous report (Fang et al., 2019) showing that the detection of large size insertion may remain a challenge for alignment-based SV callers.

#### Resource Usage

**FIGURE 4:**
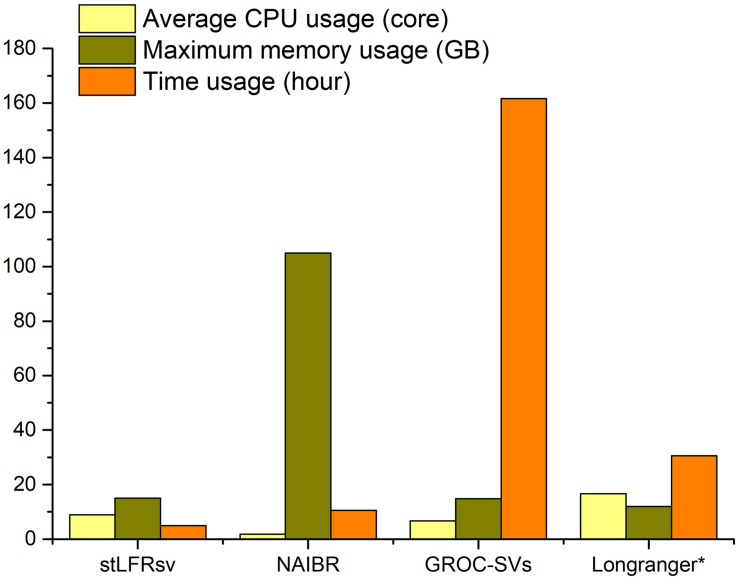
The resource usage of four pipelines when processing 100× stLFR reads of HG002 with the given parameters in Supplementary Table 1. *The resource usage of Long Ranger is estimated by the log file because it is a fully integrated functional pipeline.

The resource utilization of these four callers was collected by the Linux system tool “time” (Figure 4), and all tests were performed on a workstation with 48 CPU cores and 256 GB memory. GROC-SVs ran very slowly because of massive assembly operations. NAIBR showed an extremely high memory consumption with a low CPU load. Benefitting from the algorithm focusing only on the sub-fragment divided by large gaps, stLFRsv achieved the best performance with regard to time and memory usage while taking full advantage of the multi-core CPU.

### 10× Genomics Linked-Reads Data of HG001

The Linked-Reads data of HG001 downloaded from 10× Genomics official website was tested on all four co-barcoded read-based SV callers (for stLFRsv, the 10× Genomics barcode BX tag was converted to an stLFR-formatted barcode). Because there is not a well-characterized GIAB call set for HG001, only the large SVs were compared among the call sets and with the 10× Genomics SV results on the website.

**FIGURE 5:**
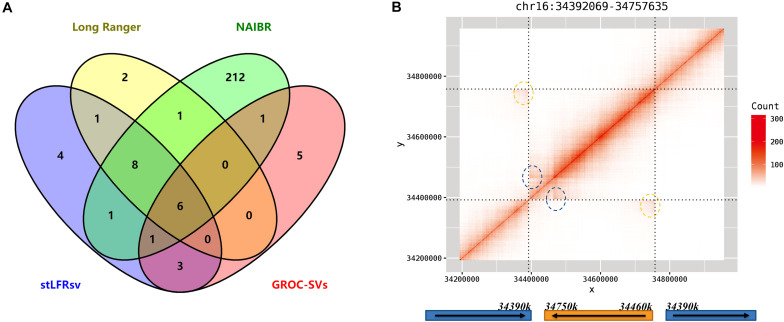
**(A)** Venn diagram of detected deletions from four structural variant (SV) callers in HG001. **(B)** The heatmap and structure for a complex inversion in HG001. The signs of two breakpoints are marked by blue and yellow circles in the heatmap.

There are 34 reliable large SVs in 10× Genomics Long Ranger call set, comprising 18 deletions, 12 duplications, and 4 inversions. To validate these SVs, they were checked by the heatmaps of co-barcode distribution manually and individually. The results are shown in Figure 5A and Supplementary Table 7. The performance of the four SV detectors on Linked-Reads data is consistent with that on stLFR data. stLFRsv has the highest consistency with each of the other three call sets. NAIBR presents the most SVs not detected by any other caller, and GROC-SVs has the least common SVs. Four deletions only detected by stLFRsv were all marked “COMMON” and also found in low-quality results in the Long Ranger call set. As for the duplications, only four duplications were confirmed as reliable variants, and they were all detected by read depth information without SV breakpoint details. The other three call sets provide minimal support for these duplications. All four inversions were detected by stLFRsv, three of which were marked “COMMON” and also found in HG002 results. The remaining inversion is a “DUP-INV” complex SV found only in HG001. As shown in Figure 5B, a DNA fragment was duplicated and inversely inserted into another genomic position of the same chromosome. Both breakpoints were detected by stLFRsv, whereas only one was reported by Long Ranger.

### 10× Genomics Linked-Reads Data of HX1

A Chinese individual, HX1, was studied and sequenced in several investigations (Shi et al., 2016; Fang et al., 2019), and a reliable SV call set of HX1 was established by SMRT-SV (Audano et al., 2019), a widely used long-read SV caller. Thus, stLFRsv was also tested on the Linked-Reads data of HX1. As stated in a previous report (Fang et al., 2019), duplications were barely detected by co-barcoded reads or long reads, and thus we only focused on deletions. The SMRT-SV call set has 16 large deletions (>10 kbp), 10 of which were detected by stLFRsv (Supplementary Table 8). The failure to detect six deletions may be the result of imprecise SV positions by stLFRsv. In other words, the size of these six deletions reported by stLFRsv may be smaller than 10 kbp, and they were accordingly found in the intermediate result file. Another deletion at Chr2:111,153,548–Chr2:111,198,923, which was missed by SMRT-SV but validated by a previous report (Fang et al., 2019), was also detected by stLFRsv (Supplementary Figure 3C).

## Discussion

We present stLFRsv, a co-barcoded read-based structural variation detector that identifies large variants with far fewer false positives than alignment-based detectors using either short reads or long reads. stLFRsv also shows the best computational performance among co-barcoded read-based SV callers. When combined with a standard short read variation caller, stLFRsv can exploit the co-barcoded reads to reveal the full spectrum of genome polymorphism. Although stLFR has decreased the average number of DNA fragments sharing the same barcode to nearly 1 and increased the coverage in “BAD” genome regions to a certain degree, co-barcoded reads have limited resolution for structural variation calling because paired reads for a long fragment only partially cover the whole sequence with unknown order and intervening distance. In contrast, the performance of single-molecule sequencing long reads has been increasing. In spite of this, discovering both base-level and very-large-scale variants simultaneously using co-barcoded sequencing technology will be promising for some clinical applications especially with lower cost and decreased turnaround time. Moreover, the greater length of DNA fragments for co-barcoded sequencing compared with single-molecule sequencing has the potential to span larger repeat regions and catch SVs missed by real long reads.

Larger variants other than deletions and insertions are needed to assess variation detection by co-barcoded reads. There are three main aspects for our future research. First, we are analyzing co-barcoded reads for clinical samples to find pathogenic balanced/unbalanced translocations, deletions, duplications, and more complex structures. This technique is likely to provide a more precise description of such variants compared with current clinical practices by identifying more reliable breakpoints. Second, another clinical application is to associate a genetic defect with nearby alleles using co-barcoded reads, which can provide an inference as to whether an infant inherited a defect through prenatal cell-free DNA sequencing by detecting associated nearby alleles. This application benefits from the outstanding phasing ability of co-barcoded reads. The final element of future work is to add a local assembly module to enhance small variation detection (in the range of 50 bp–1 kbp).

With a cost slightly higher than standard short reads, co-barcoded reads are able to reveal much more useful information for the underlying genomes.

## Data Availability Statement

The original contributions presented in the study are included in the article/Supplementary Material, further inquiries can be directed to the corresponding author/s.

## Ethics Statement

The studies involving human participants were reviewed and approved by the Institutional Review Board of BGI. The patients/participants provided their written informed consent to participate in this study.

## Author Contributions

PL and OW conducted the co-barcoded reads library construction. JG, CS, and XC developed the pipeline and analyzed the data. XX and HY designed the research. WZ supervised and coordinated all aspects of the project. HZ wrote the manuscript. All authors revised, read, and approved the final manuscript.

## Conflict of Interest

The authors declare that the research was conducted in the absence of any commercial or financial relationships that could be constructed as a potential conflict of interest. The handling editor declared a past co-authorship with the authors OW, HY, XX, and WZ.

## Footnotes

1 https://github.com/spiralgenetics/truvari

## Supplementary Material

Supplementary Figure 1 Basic data profile distribution for stLFR co-barcoded reads and long reads of HG002. **(A)** Read pairs per barcode. **(B)** Weighted fragment length for co-barcoded reads. **(C)** Number of fragments per barcode. **(D)** Long read length. **(E)** Long read percent identity.

Supplementary Figure 2 An example of empirical gap size distribution of stLFR co-barcoded reads. Different selected *P*s help to decide the parameters used in the pipeline.

Supplementary Figure 3 Heatmaps of **(A)** Three false negative large deletions in HG002. **(B)** A false positive deletion by different aligners in HG002. **(C)** A validated deletion detected by stLFRsv in HX1.

Supplementary Figure 4 Weighted fragment length distribution for different HG002 stLFR libraries.
