# Supplementary figures and images for: stLFRsv: A Germline Structural Variant Analysis Pipeline Using Co-barcoded Reads
Source: Front Genet. 2021 Mar 18;12:636239. doi: 10.3389/fgene.2021.636239 (PMC8012683; doi:10.3389/fgene.2021.636239)

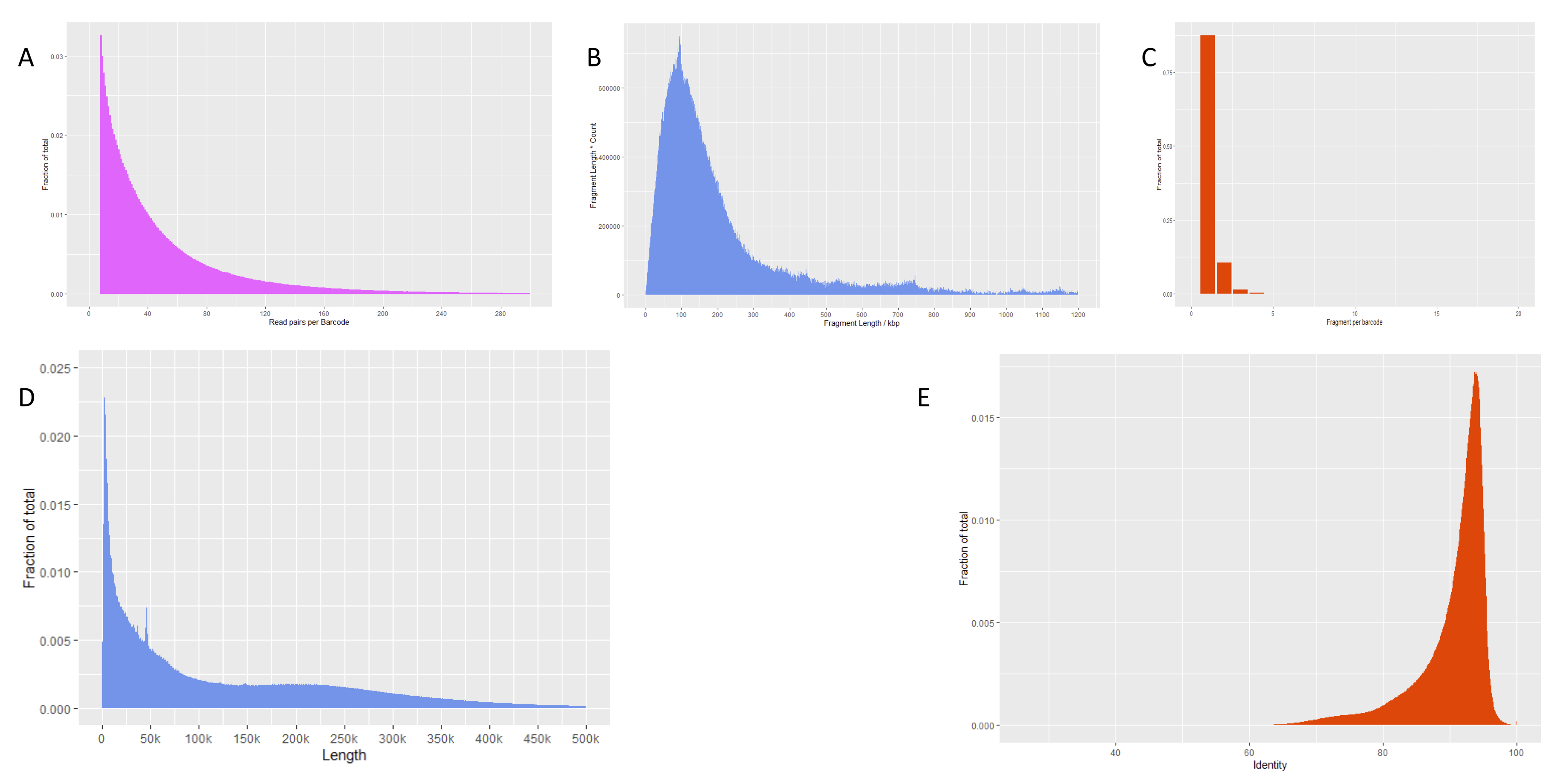

Supplement: Supplementary Figure 1 — Basic data profile distribution for stLFR co-barcoded reads and long reads of HG002. (A) Read pairs per barcode. (B) Weighted fragment length for co-barcoded reads. (C) Number of fragments per barcode. (D) Long read length. (E) Long read percent identity. [file Image_1.TIF]

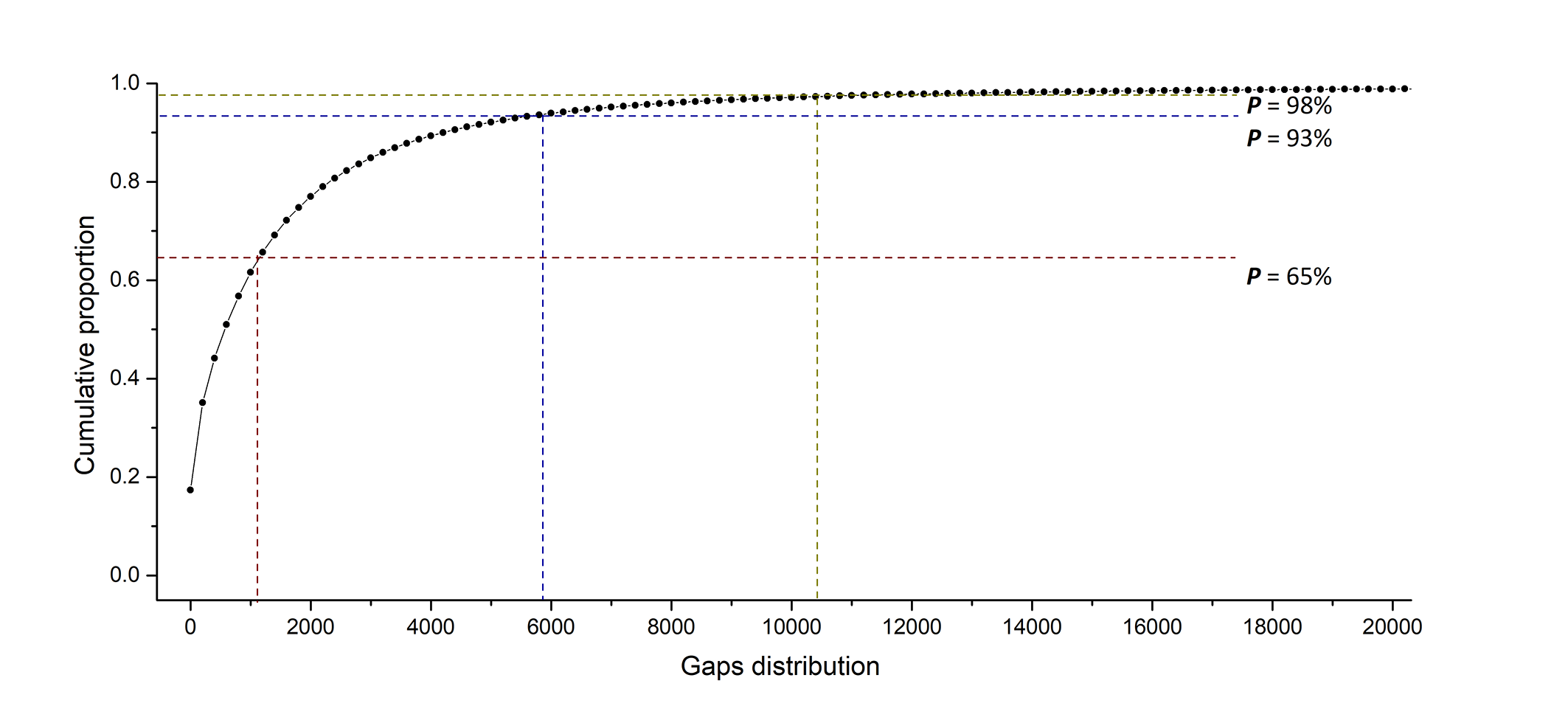

Supplement: Supplementary Figure 2 — An example of empirical gap size distribution of stLFR co-barcoded reads. Different selected Ps help to decide the parameters used in the pipeline. [file Image_2.TIF]

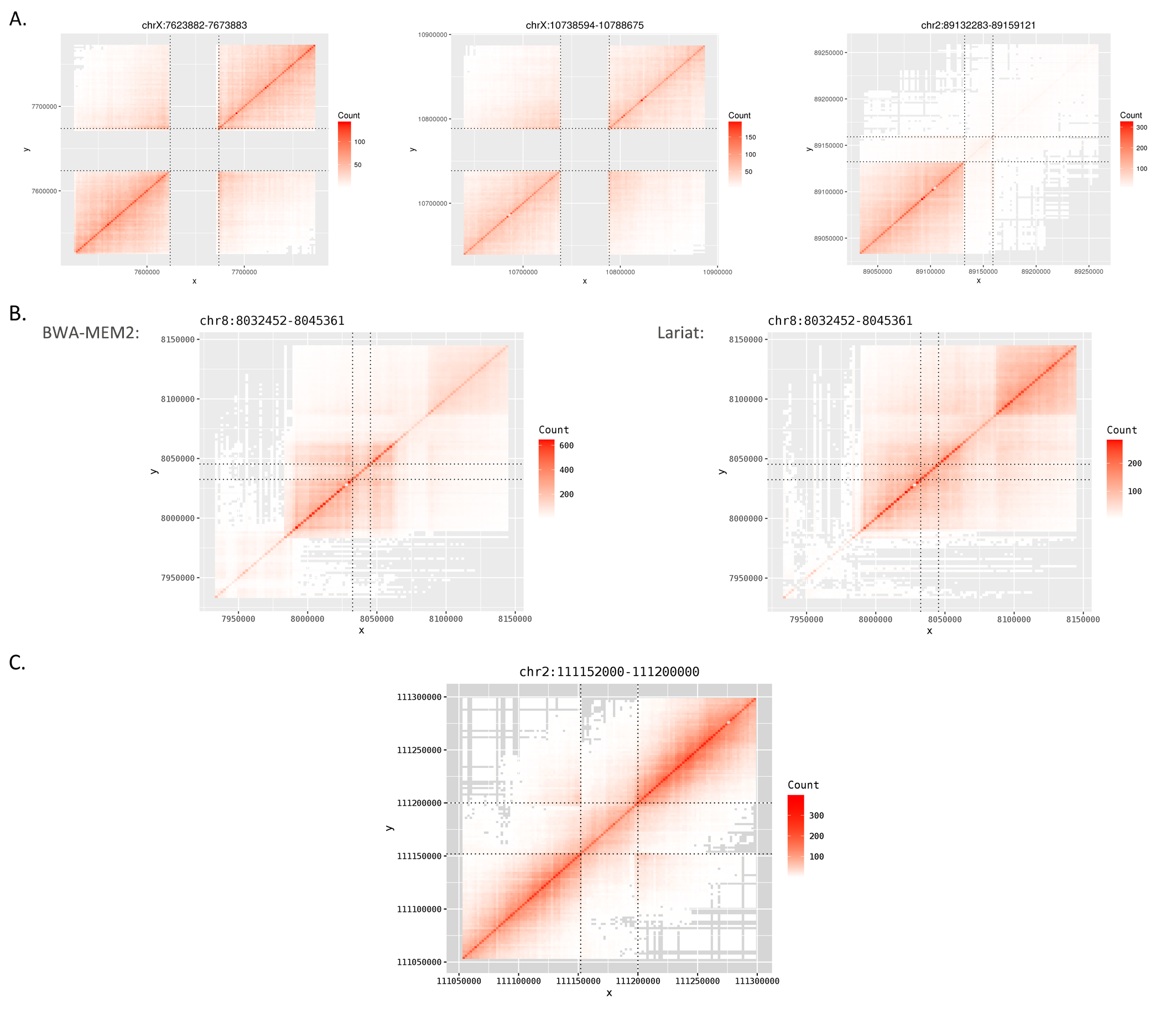

Supplement: Supplementary Figure 3 — Heatmaps of (A) Three false negative large deletions in HG002. (B) A false positive deletion by different aligners in HG002. (C) A validated deletion detected by stLFRsv in HX1. [file Image_3.TIF]

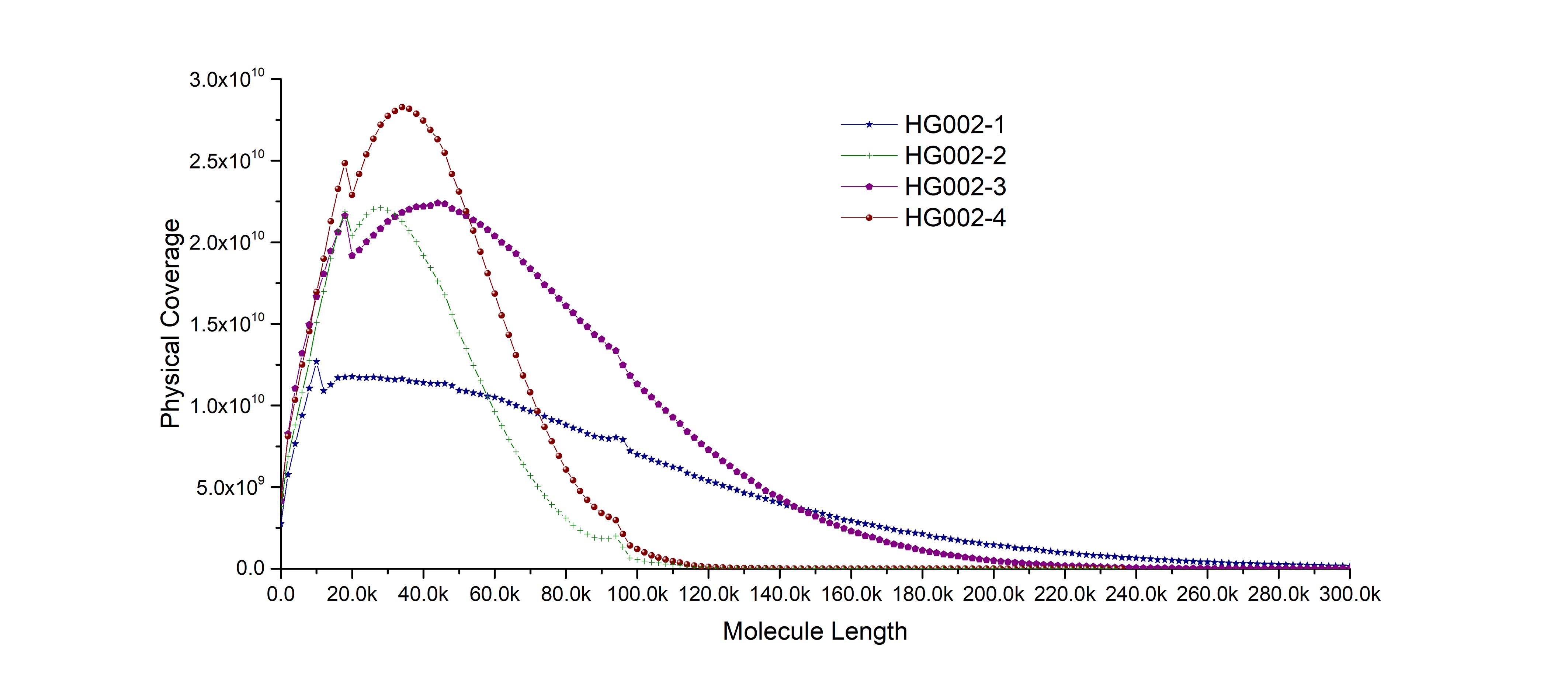

Supplement: Supplementary Figure 4 — Weighted fragment length distribution for different HG002 stLFR libraries. [file Image_4.TIF]
